# Synthesization, Characterization, and *in Vitro* Evaluation of Cytotoxicity of Biomaterials Based on Halloysite Nanotubes

**DOI:** 10.3390/ma7127770

**Published:** 2014-12-04

**Authors:** Antonio Sánchez-Fernández, Laura Peña-Parás, Román Vidaltamayo, Rodrigo Cué-Sampedro, Ana Mendoza-Martínez, Viviana C. Zomosa-Signoret, Ana M. Rivas-Estilla, Paulina Riojas

**Affiliations:** 1 Instituto Tecnológico de Estudios Superiores de Monterrey, Eugenio Garza Sada 2501, Col. Tecnológico, 64849 Monterrey, NL, Mexico; E-Mails: asanfer@itesm.mx (A.S.-F.); rodrigo.cue.sampedro@itesm.mx (R.C.-S.); 2 Departamento de Ingeniería, Universidad de Monterrey, Ave. Morones Prieto 4500 Pte., Col. Jesús M. Garza, 66238 San Pedro Garza García, NL, Mexico; E-Mails: ana.mendoza@udem.edu (A.M.-M.); paulina.riojas@udem.edu (P.R.); 3 Departamento de Ciencias Básicas, Universidad de Monterrey, Ave. Morones Prieto 4500 Pte., Col. Jesús M. Garza, 66238 San Pedro Garza García, NL, Mexico; E-Mails: roman.vidaltamayo@udem.edu (R.V.); amrivas1@yahoo.ca (A.M.R.-E.); 4 Departamento de Bioquímica y Medicina Molecular, Facultad de Medicina, Universidad Autónoma de Nuevo León, Jesús Aguirre Pequeño esq. Av. Madero, 64460 Monterrey, NL, Mexico; E-Mail: vivizomo@gmail.com

**Keywords:** HNTs, organosilanes, characterization, functionalization, cytotoxicity

## Abstract

Halloysite is an aluminosilicate clay that has been widely used for controlled drug delivery, immobilization of enzymes, and for the capture of circulating tumor cells (CTCs). Surface modification of halloysite by organosilanes has been explored to improve their properties. In this study halloysite clay nanotubes (HNTs) were functionalized by two different organosilanes: Trimethoxy(propyl)silane (TMPS), and Triethoxy(octyl)silane (EOS). Untreated and modified samples were characterized by scanning electron microscopy (SEM), X-ray diffractometry (XRD), thermogravimetrical analysis (TGA), and Fourier transform infrared spectroscopy (FTIR). Results showed a strong interaction of organosilanes with the chemical groups present in HNTs. Biocompatibility and cytotoxicity of these nanomaterials were determined using C6 rat glioblastoma cells. Our results indicate that prior to functionalization, HNTs show a high biocompatibility and low cytotoxicity. However, HNTs functionalized with EOS and TMPS showed high cytotoxicity by inducing apoptosis. These results allow the identification of potential applications in biomedical areas for HNTs.

## 1. Introduction

Halloysite nanotubes (HNTs) have been recently used due to their unique properties such as their hollow tubular structure, high surface area, surface characteristics, and high biocompatibility [1,2,3,4,5,6]. HNT tubules consist of a two layered aluminosilicate clay (Al_2_Si_2_O_5_(OH)_4_∙2H_2_O) with an external diameter of 50–80 nm, lumen diameter of 10–15 nm, and length of 1000 nm [7]. The tubes have multilayer walls with positively charged Al–OH functional groups on the inner surface, and with negatively charged Si–OH functional groups on the outer surface [8]. These characteristics make HNTs a great absorbent for both cationic and anionic molecules [1,2,3,4,5]. Furthermore, their nanosized lumen enables entrapping a range of active agents such as macromolecules, and proteins [9]. The advantages of these nanoparticles compared to carbon nanotubes, which have been extensively studied for a wide range of applications, are that HNTs are not toxic for the human body nor hazardous for the environment, and have lower manufacturing costs [7].

For these reasons HNTs have been studied for diverse biomedical applications, including inexpensive drug encapsulation [8,10,11,12], as a template or nanoreactor for biocatalyst [13], as well as for use in personal care and cosmetics [14]. Zhai *et al.* [15] demonstrated that HNTs can also be used to immobilize enzymes, such as the α-amylase and urease with the objective of extending their catalytic lifetime. This study showed that the immobilized enzymes had more than 90% of activity due to the presence of HNTs after 15 days. Moreover, HNTs have shown to be successful for enhanced isolation and capture of circulating tumor cells (CTCs) in blood [16,17,18]. For example, Hughes *et al.* [17] designed a microscale flow device for isolating CTCs where the addition of a HNT coating improved capture purities. Targeted drug delivery to CTCs has also being achieved with HNT-liposome coated surfaces [19,20], thus reducing the probability of metastasis and chemotherapeutic dosages, as shown by Mitchell *et al.* [20].

HNTs may be modified either with salts or organosilanes [21] in order to improve the interaction with polymer matrices [22,23,24]. It has been proved that coating HNTs with organic molecules on its surface may reduce agglomeration due to its interaction with organic media. According to Gironès *et al.* [25], organosilanes are widely used because of their low cost and availability. Yuan *et al.* [8] modified HNTs with γ-Aminopropyltriethoxysilane (APTES) by direct grafting of the organosilane onto the surface hydroxyl groups. Similarly, Shi *et al.* [26] functionalized HNTs with APTES to use them as carriers of therapeutic gene antisense oligodeoxynucleotides (ASODNS). Due to the large aspect ratio, good biocompatibility, and high mechanical strength of the modified HNTs, it was demonstrated that these nanotubes are a promising vector for gene therapy applications.

In this study HNTs were functionalized with two different organosilanes: Trimethoxy(propyl)silane (TMPS), and Triethoxy(octyl)silane (EOS), and characterized by scanning electron microscopy (SEM), X-ray diffractometry (XRD), thermogravimetrical analysis (TGA), and Fourier transform infrared spectroscopy (FTIR). The biocompatibility and cytotoxicity of these materials was determined using C6 rat glioblastoma cell cultures. SEM, FTIR, TGA, and cytotoxicity results from the untreated and modified HNTs were previously presented at the First International Electronic Conference on Materials [27].

## 2. Experimental Method

### 2.1. Materials

HNTs, Trimethoxy(propyl)silane (TMPS), and Triethoxy(octyl)silane (EOS) were purchased from Sigma-Aldrich (St. Louis, MO, USA). Analytical grade acetone from CTR (Monterrey, Mexico) was also used for sample preparation. Collagen from calf skin, acetic acid, and Phosphate buffered saline (PBS) were obtained from Sigma-Aldrich. Dulbecco’s Modified Eagle’s medium (DMEM) medium, fetal bovine serum (FBS), l-glutamine, trypsin, and penicillin-streptomycin are from Life Technologies (New York, NY, USA).

### 2.2. Functionalization of HNTs

HNTs were functionalized with two different organosilanes, namely Trimethoxy(propyl)silane, and Triethoxy(octyl)silane. Functionalization was carried out by mixing in a flask ball 10 g of HNTs, 50 mL of acetone, and 2 mL of organosilane material. The samples were heated at 50 °C for 48 h in order to eliminate unreacted materials. Finally, the suspensions were vacuum filtered to obtain a solid phase.

### 2.3. Characterization Methods

FTIR-ATR spectra were obtained with a Perkin Elmer SPECTRUM 400 spectrometer (Waltham, MA, USA) using a ZnSe trapezoidal shaped ATR element. Sample spectrum and background were acquired with the coated ATR element and the clean ATR element, respectively. The spectra were acquired with a resolution of 4 cm^−1^ and 16 scans. An FEI Nova NanoSEM 200 scanning electron microscope (SEM) (Hillsboro, OR, USA), with an acceleration voltage of 15 kV, and secondary electron detector under vacuum, was used to characterize the morphology of the untreated and functionalized HNTs. The Energy-dispersive X-ray spectroscopy (EDS) elemental analysis was performed using an INCA X-Sight (Abingdon, UK). XRD analysis was performed by an Empyrean PANalytical diffractometer (Boulder, CO, USA) with an X’Cellerator detector in a continuous mode scanning with a start angle of 30.000°, and end angle of 100.000°, a step size of 0.02, and time per step of 1 s. An X-ray tube copper wavelength (λ) of 1.5405 was used, at a voltage of 45 kV, and current of 40 mA. Decomposition temperatures of samples were determined by thermogravimetrical analysis (TGA) with a TA Instruments SDT Q600 (New Castle, DE, USA). All samples were heated al 10 °C/min from room temperature (25 °C) to 850 °C under 100 mL/min nitrogen purge.

### 2.4. Cell Culture

C6 Rat glioblastoma cells were obtained from American Type Culture Collection (cat. no. CCL-107, Manassas, VA, USA). The cell line was cultured at 37 °C under recommended conditions in tissue culture-treated plastic dishes (Corning) with DMEM medium supplemented with 10% FBS, 2 mM l-glutamine, 100 U/mL of penicillin, and 100 μg/mL streptomycin.

### 2.5. Cytotoxicity Analysis

Materials were autoclaved for 15 min at 100 °C, followed by a 30 min drying cycle. Then, they were left to cool, and stored at room temperature (25 °C).

Cell culture-treated 96-well fluorescence microplates (Corning, cat. no 3916, Corning, NY, USA) were covered with a 0.05 mg/mL solution of collagen in 0.1 M of acetic acid overnight. After this incubation step, the wells were washed 3 times with phosphate buffered saline solution (PBS, 0.1 M phosphate buffer, 0.9% *w*/*v* sodium chloride, pH 7.2). After washing, 20,000 C6 cells were plated per well, and incubated overnight to reach 60% confluence. At this moment, the different treatments (see Table 1) were administered, and cells were incubated overnight. Cytotoxicity and apoptosis assays where then performed using the ApoTox-Glo Triplex assay (Promega, cat. no. G6320, Fitchburg, WI, USA) following the manufacturer’s recommendations.

Assays were read in a GloMax automated plate fluorometer (Promega) at 402 nm/505 nm (excitation/emission) for cell viability, and at 485 nm/520 nm for cytotoxicity assessments.

**Table 1:** Experimental groups used to evaluate cytotoxicity of halloysite clay nanotubes (HNTs) derivatives.

Reaction	Cells	Collagen	HNTs	HNTs–TMPS	HNTS–EOS
Control	X	–	–	–	–
Collagen	X	X	–	–	–
HNTs/Collagen	X	X	0.05%	–	–
HNTs–TMPS/Collagen	X	X	–	0.05%	–
HNTs–EOS/Collagen	X	X	–	–	0.05%

X: indicate the presence of a component; –: indicate absence.

A control group was included to evaluate base-line cell mortality at 80% confluence. Collagen was included in all the groups exposed to nanomaterial derivatives. Nanomaterials were first re-suspended as colloidal suspensions at 5% (*w*/*v*) in cell culture medium, and then stirred prior to diluting to final concentrations of 500 µg/mL in fresh medium before adding the mixture to the cell cultures.

The results obtained from cytotoxicity tests were analyzed using Sigma Stat 3.5 program (SYSTAT, San Jose, CA, USA). Data were statistically analyzed with a one-way ANOVA test, and Bonferroni multiple comparisons post-hoc test.

## 3. Results and Discussion

### 3.1. Characterization Results

**Figure 1:**
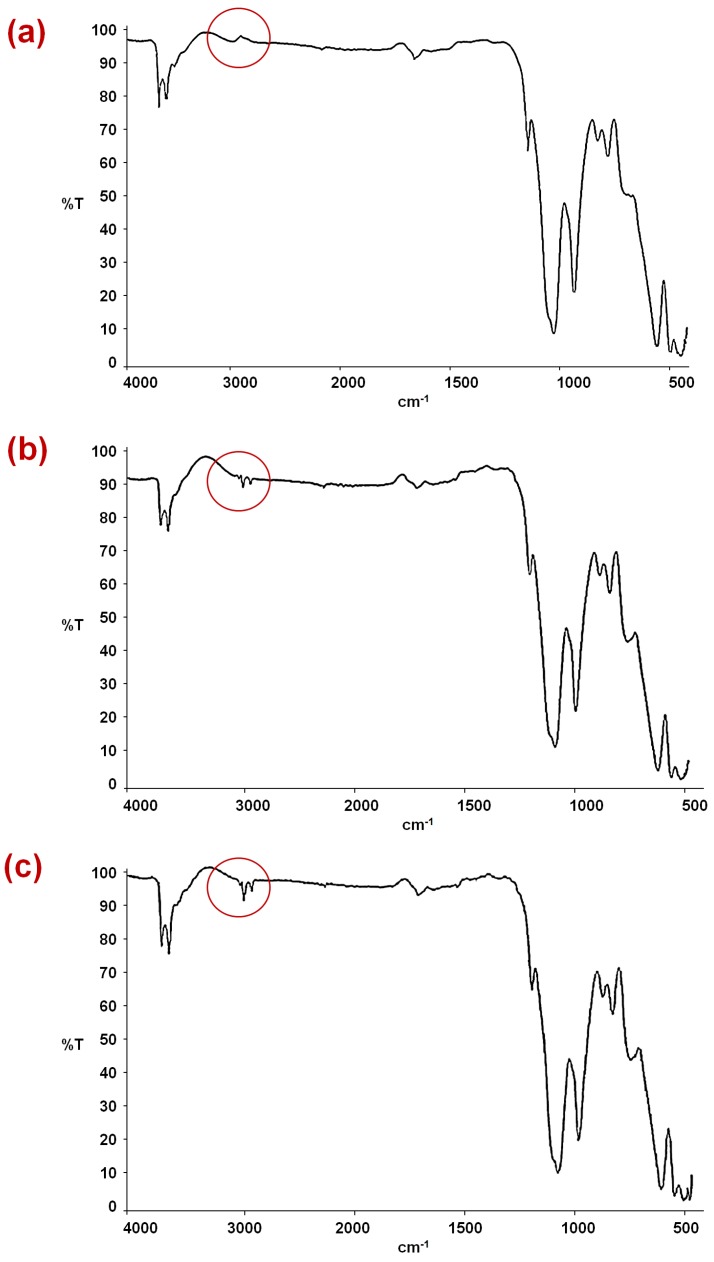
FTIR images of (**a**) HNTs; (**b**) HNTs–Trimethoxy(propyl)silane (TMPS); (**c**) HNTs–Triethoxy(octyl)silane (EOS).

Figure 1 depicts the FTIR spectrums of HNTs, HNTs–TMPS, and HNTs–EOS. The vibrational mode at 2924 cm^−1^ on Figure 1b and Figure 1c corresponds to the methyl and methylene groups (area marked with a circle), that are not present in HNTs, confirming the chemical interaction with Al–O or Si–O functional groups of HNTs. The HNTs–TMPS and HNTs–EOS show some signals due to HNTs, such as the deformations of Al–O–Si and Si–O–Si at 536 and 462 cm^−1^, respectively, the O–H groups of the inner hydroxyl groups at 909 cm^−1^. Compared to HNTs no other characteristic signals are detected in HNTs–TMPS or in HNTs–EOS.

**Figure 2:**
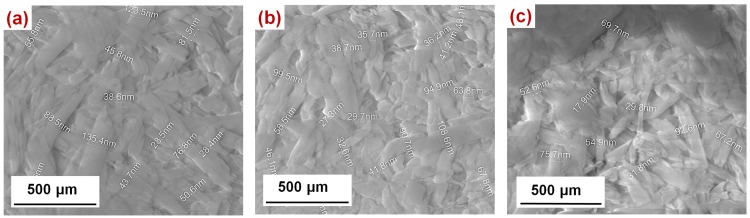
SEM images of (**a**) HNTs; (**b**) HNTs–TMPS; (**c**) HNTs–EOS.

**Figure 3:**
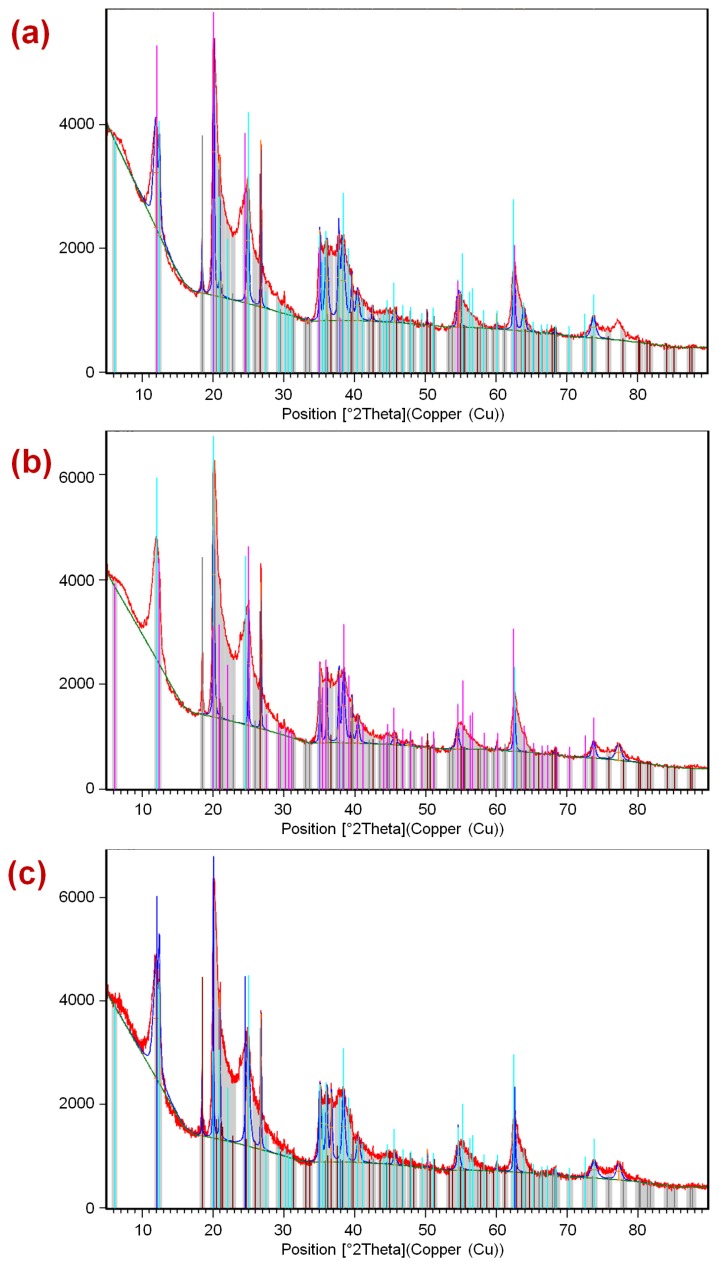
Diffractograms of (**a**) HNTs; (**b**) HNTs–TMPS; (**c**) HNTs–EOS.

SEM images of HNTs, with a diameter of 20–135 nm and various lengths, are shown in Figure 2. Micrographs show that HNTs (Figure 2a), HNTs–TMPS (Figure 2b) and HNTs–EOS (Figure 2c) have no significant structural changes, showing only particles formed in fibers or wires with nanometric sizes. This is confirmed by XRD diffractograms, as shown in Figure 3.

Table 2 depicts the EDS analysis results in wt%. This test proved that the major constituents for the HNTs, HNTs–TMPS, and HNTs–EOS were Al, Si, and O. The carbon content is not included because the sample particles were supported on a carbon ribbon. 

**Table 2:** Energy-dispersive X-ray spectroscopy (EDS) analysis results.

Material	O (wt%)	Al (wt%)	Si (wt%)
HNTs	61.84	18.48	19.69
HNTs–TMPS	60.85	19.22	19.94
HNTs–EOS	62.57	19.92	17.51

Figure 3 presents the diffractograms obtained by XRD of the three samples (HNTs, HNTs–TMPS, and HNTs–EOS), showing no significant differences between them. In the three solids the following phases were identified: Halloysite-7A, and Halloysite-14A. Furthermore, the phases of SiO_2_ and Al(OH)_3_ were indexed. The three solids are composed basically of two polymorphs of the bilaminar clay (1:1) dioctahedral Halloysite, in the presence of SiO_2_ and Al(OH)_3_. Thus, it is concluded that during the process of functionalization, a modification of the chemical structure of Halloysite is not appreciated.

Table 3 shows the weight loss (%) at 200–320 °C for functionalized and untreated HNTs obtained by TGA. The higher weight loss shown from HNTs–EOS compared to HNTs–TMPS is consistent with the higher hydrocarbon chain of the octyl group from EOS. This also supports the strong chemical interaction of the organosilanes with the Si–O and Al–O groups of HNTs. The remaining materials at 800 °C (~81%) are oxides of aluminum and silicon, compounds present in HNTs.

**Table 3:** Weight loss by thermogravimetrical analysis (TGA) of HNTs and functionalized HNTs.

Sample	Weight Loss in TGA, 200–320 °C (%)	Difference Relative to HNTs (%)
HNTs	1.86	–
HNTs–TMPS	1.94	0.08
HNTs–EOS	2.04	0.18

### 3.2. Cytotoxicity Results

**Figure 4:**
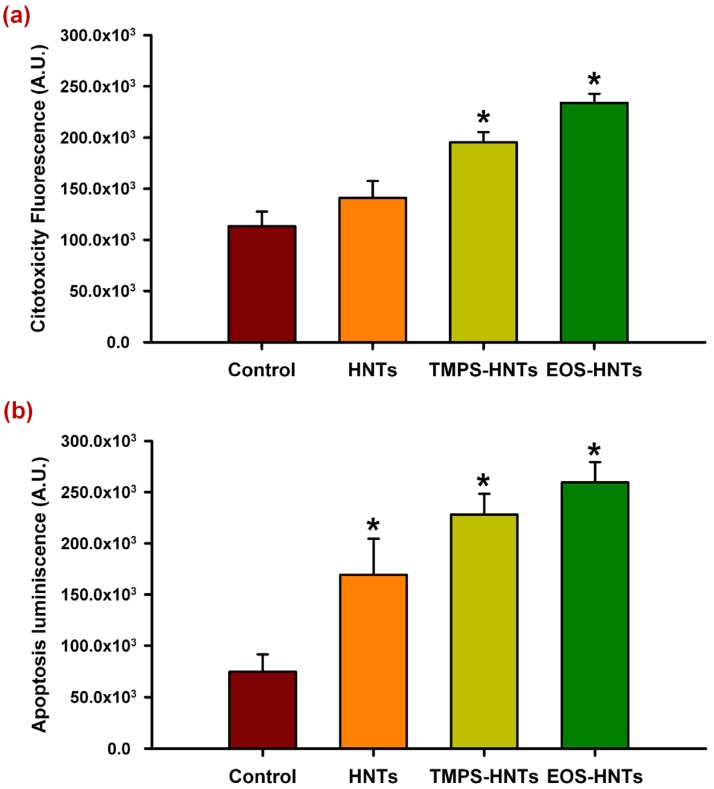
Cytotoxic and apoptotic effects of HNTs derivatives. Organosilane derivatives of HNTs (TMPS, EOS) increase cell mortality after 24 h exposure (**a**) These cytotoxic effect is mediated by a pro-apoptotic activity; (**b**) Unmodified HNTs do not show a statistically significant increase in cytotoxicity although they do increase apoptosis, albeit at a lower level. * denotes *p* < 0.01.

Figure 4 shows the cytotoxic and apoptotic effects of unmodified and organosilane functionalized HNTs.

Unmodified HNTs do not appear to have cytotoxic effects after a 24 h exposure period in C6 glioma cell cultures. However, TMPS- and EOS-functionalized HNTs increased cell mortality after incubating under the same conditions (Figure 4a). These cytotoxic effects are due to an increase in apoptosis, which is also observed in cultures exposed to unmodified HNTs (Figure 4b). It is possible that HNTs activate pro-apoptotic signaling in C6 cells, which can be enhanced by organosilane functionalization, leading to increased cell death after 24 h only by exposure to these derivatives.

This cytotoxic effect could take longer exposure times to be appreciated with unmodified HNTs and it is necessary to perform further experiments to determine if this is the case. Moreover, evaluation on other cell types is necessary in order to determine whether induction of apoptosis is cell-type specific or not.

Previous studies by Vergaro *et al.* [6] showed that both non-functionalized HNTs and APTES-functionalized HNTs appear to be well tolerated by breast cancer cell (MCF-7) cultures on concentrations up to 75 μg/mL, with a decrease in cell vitality at higher concentrations. Our results show that at 500 μg/mL organosilane functionalization of HNTs increase pro-apoptotic activity that leads to enhanced cytotoxic effects, however non-functionalized HNTs at the same concentration appear to be biocompatible. Differences in the cytotoxicity observed in our study could be due to the effect of the chemical nature of the organosilane derivatives we used, or on the other hand, from the different approaches used to determine cytotoxicity: Vergaro *et al.* [6] used the 3-(4,5-dimethylthiazol-2-yl)-2,5-diphenyltetrazolium bromide (MTT) reduction assay, which is dependent on cell metabolism. Reduction of MTT can be altered by a number of factors that do not lead to apoptosis, including, but not limited to, disruptors of mitochondrial function and inhibitors of cell proliferation with no cytotoxic effects [28], while our assays determine the release of cytosolic proteases and the level of activation of the caspase pathway [29]. It would be interesting to evaluate if further functionalization with large biomolecules or therapeutic drugs could revert the cytotoxic activity of the organosilane derivatives.

## 4. Conclusions 

Functionalization of HNTs was achieved by modification with TMPS and EOS organosilanes. Characterization by FTIR and TGA showed the strong interaction of organosilanes with the chemical groups present in HNTs. This functionalization may be useful to improve the properties of HNTs for several applications, including drug encapsulation and delivery, biocatalysis, and for nanocomposites with enhanced mechanical properties. Cytotoxicity of untreated and functionalized HNTs was characterized. Results showed that at the selected concentration HNTs showed to be a highly biocompatible material, however, functionalization by selected organosilanes exhibited high cytotoxicity, inducing cell death by apoptosis.
